# Investigation of temperature variations on a Class-E inverter and proposing a compensation circuit to prevent harmful effects on biomedical implants

**DOI:** 10.1038/s41598-023-31076-y

**Published:** 2023-03-10

**Authors:** Mehrnaz Khodadoost, Mohsen Hayati, Hamed Abbasi

**Affiliations:** 1 Electrical Engineering Department, Faculty of Engineering, Razi University, Kermanshah, 67149-67346 Iran; 2 Department of Electrical Engineering, Kermanshah Branch, Islamic Azad University, Kermanshah, Iran

**Keywords:** Electrical and electronic engineering, Design, synthesis and processing

## Abstract

In this paper, a Class-E inverter and a thermal compensation circuit for wireless power transmission in biomedical implants are designed, simulated, and fabricated. In the analysis of the Class-E inverter, the voltage-dependent non-linearities of C_ds_, C_gd_, and R_ON_ as well as temperature-dependent non-linearity of R_ON_ of the transistor are considered simultaneously. Close agreement of theoretical, simulated and experimental results confirmed the validity of the proposed approach in taking into account these nonlinear effects. The paper investigated the effect of temperature variations on the characteristics of the inverter. Since both the output power and efficiency decrease with increasing temperature, a compensation circuit is proposed to keep them constant within a wide temperature range to enable its application as a reliable power source for medical implants in harsh environments. Simulations were performed and the results confirmed that the compensator enables significant improvements by maintaining the power and efficiency almost constant (8.46 ± 0.14 W and 90.4 ± 0.2%) within the temperature range of − 60 to 100 °C. Measurements performed at 25 °C and 80 °C with and without the compensation circuit were in good agreement with the theoretical and simulation results. The obtained measured output power and efficiency at 25 °C are equal to 7.42 W and 89.9%.

## Introduction

**Figure 1:**
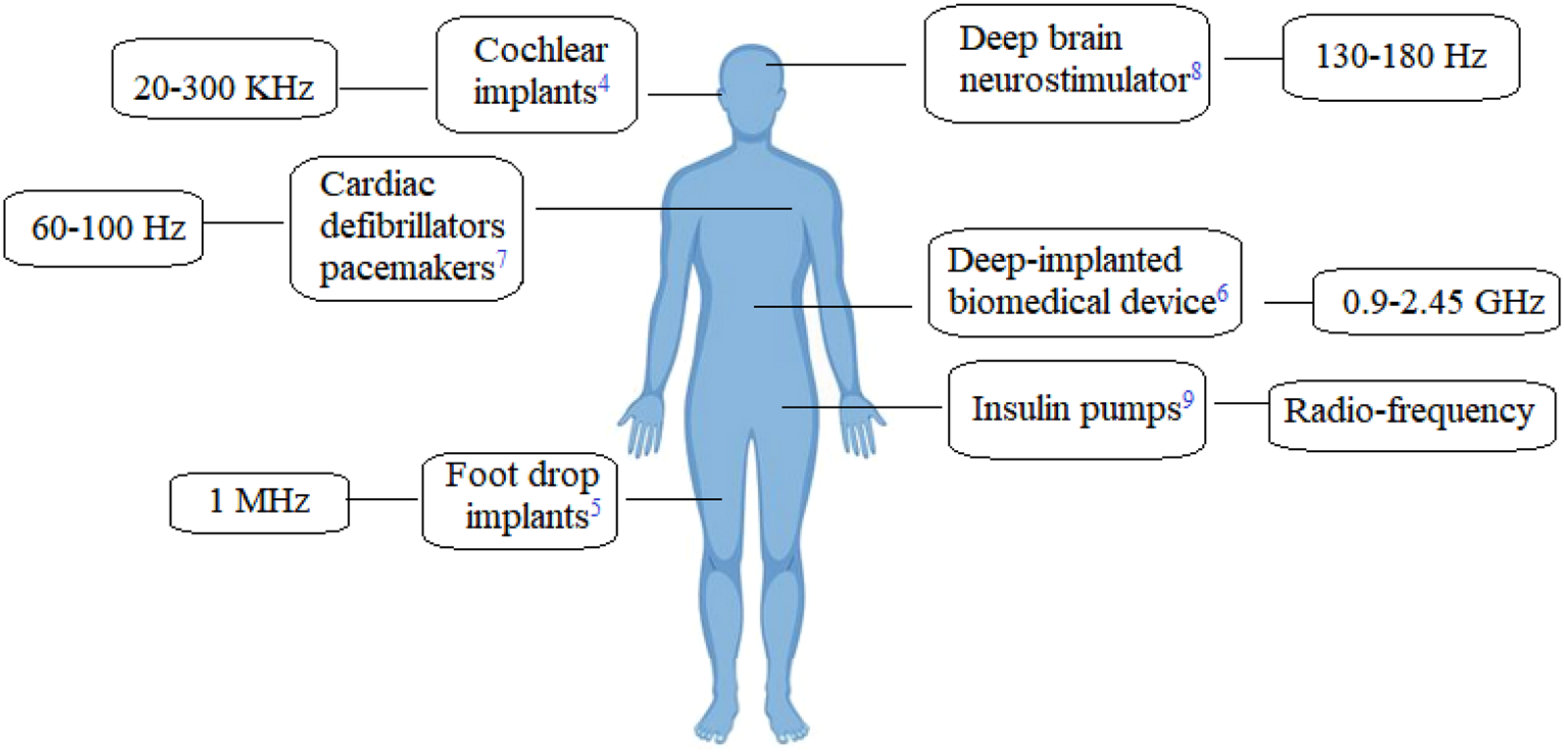
Some of the therapeutic implants which are supplied with WPT^10^.

Today, wireless technology plays an important role in the development of Health informatics. The convenience of patients under treatment, diagnosing the disease with the least side effects and the accuracy of test results are important concerns of scientists in this field. Wireless technology is used in biomedical sensors, artificial organs, and remote monitoring of the patients’ condition^1–3^. Figure 1 shows some examples of implants that integrate wireless technology for therapeutic applications. The illustrated implants work at different frequencies and are designed according to their function in different parts of the body^4–9^_._ Class-E Power Amplifier (PA) or inverter is the main component of WPT^11^. In design and analysis of Class-E power amplifier, the effects of input voltage waveforms^12^, duty ratio^13^, and parasitic linear and nonlinear capacitances^14^ have been discussed. Also, comprehensive research projects have been conducted to improve the characteristics of PAs in different classes for biomedical industry^15^ One of the important issues in the manufacture of biomedical implants is availability of reliable wireless power supplies and this paper is focused on this matter specially with regards to operation in harsh environments.

The Class-E inverter has been welcomed due to its high efficiency, circuit simplicity, and small size and therefore it has been the main element of practical circuits such as biomedical implants^16,17^, inverters^18–20^, WPT systems^21–26^, oscillators^23,28^, and light communication transmitter^29^. Due to these broad applications, a variety of techniques have also been used to design and optimize class E inverters for different WPT applications. As an example, in Inductive Power Transfer (IPT) WPT, due to the displacement of the transmitter and receiver coils, as well as the displacement of the alignment between them, the values of the output resistance and inductance of the inverter changes. To avoid the effects of these changes, frequency modulated control^24^ and load independent class E inverter^25,26^ have been presented. The load-independent Class-E inverter achieves constant output voltage, ZVS, and fixed phase shift between the driving signal and the output voltage at any load resistance without any control^24^.

Another approach is to merge Class-E PAs with other classes, which results in mixed-mode PAs such as class D_EM_^30^ and class E_M_/F_n_^31^. The robust and efficient wireless power transfer and the floating bulk technique achieved by using a power-efficient Class-E power amplifier have also been investigated^32,33^_._ In one research^34^, a study of the temperature effect on the MOSFET biased in the subthreshold region is presented. In another work two Class-E PAs with transmission line (TL), lumped elements^35^, and a Class-E PA with two cascaded transistors^36^ have been studied. The design of the Class-E PA considering the non-linear drain-source and linear gate-drain capacitances have also been studied^12,37,38^ In a recent work^39^, the temperature effect on the Class-E PA has been investigated where the effect of temperature has been compensated by DC gate-source voltage, despite its undesired influence on the transistor bias region, also only the drain-source resistor of the transistor is considered as a linear element and the effect of nonlinear capacitors is completely neglected. This is while, the need to consider these capacitors in the analysis has already been thoroughly proven^40^. Studying the effects of temperature changes on WPT transmitters while also considering the simultaneous effect of nonlinear capacitances and on-state resistance is a missing challenge of research, which is the main aim of this paper. We have then proceeded to propose a compensation circuit which does not adversely affect the bias of the MOSFET and aims to exceed the performance of the previous approach. This is done with the objective of maximizing the operating temperature range of the inverter as defined by its power level and efficiency. In this paper, it is shown that the output power and efficiency decrease with increasing temperature. The change in temperature also changes the drain-source voltage, which it may exceed the maximum withstand voltage of the transistor and as a result, the transistor can be damaged in the circuit. This condition would be worse if we had a MOSFET with a large R_ON_. In medical implants, providing a reliable power source is one of the designers’ issues. A compensation circuit is proposed in this paper to keep output power and efficiency constant within a wide temperature range in order to enable its application as a reliable power source for medical implants in harsh environments.

This paper is categorized as follows; discussion of the design block diagram and design specification, presentation of the proposed analysis for the Class E Inverter considering the voltage-dependent non-linearities of Cds, Cgd, and R_ON_ as well as temperature-dependent non-linearity of R_ON_ of the transistor and investigating temperature variation effects, introducing the class-E inverter with the proposed thermal compensation circuit, and finally presentation and discussion of the measurement results and a conclusion.

## Proposed methodology

### Design block diagram and design specification

**Figure 2:**
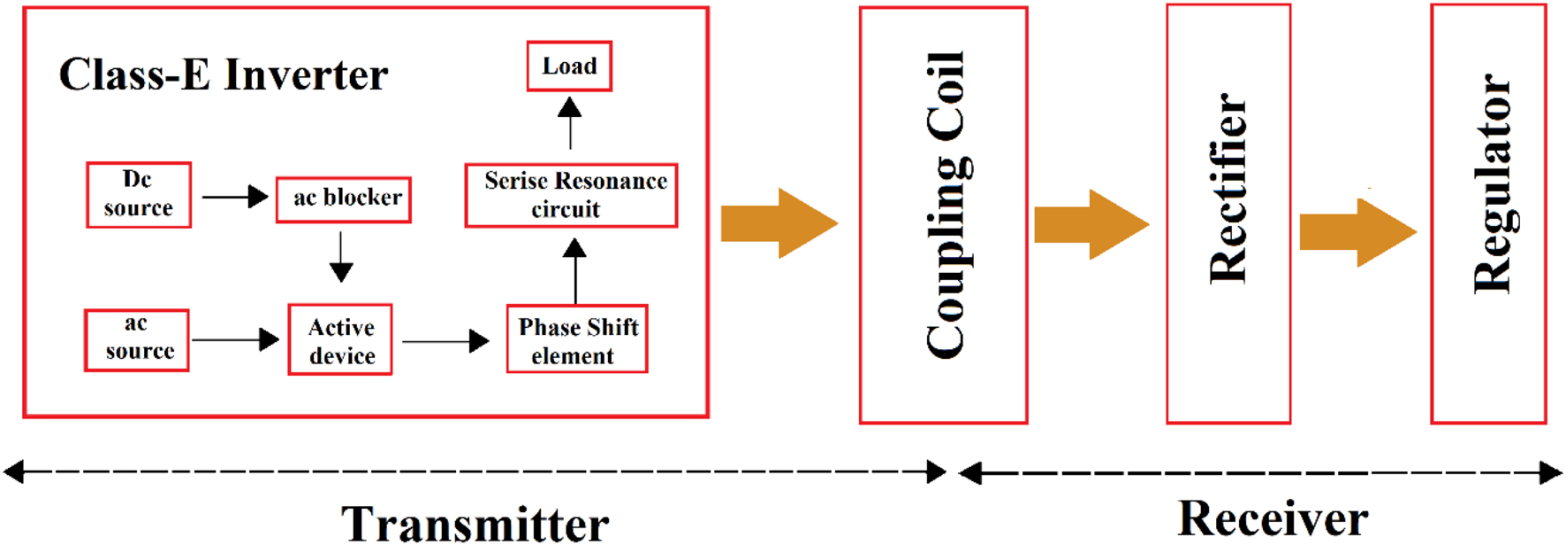
The proposed block diagram of a WPT in biomedical implants.

**Figure 3:**
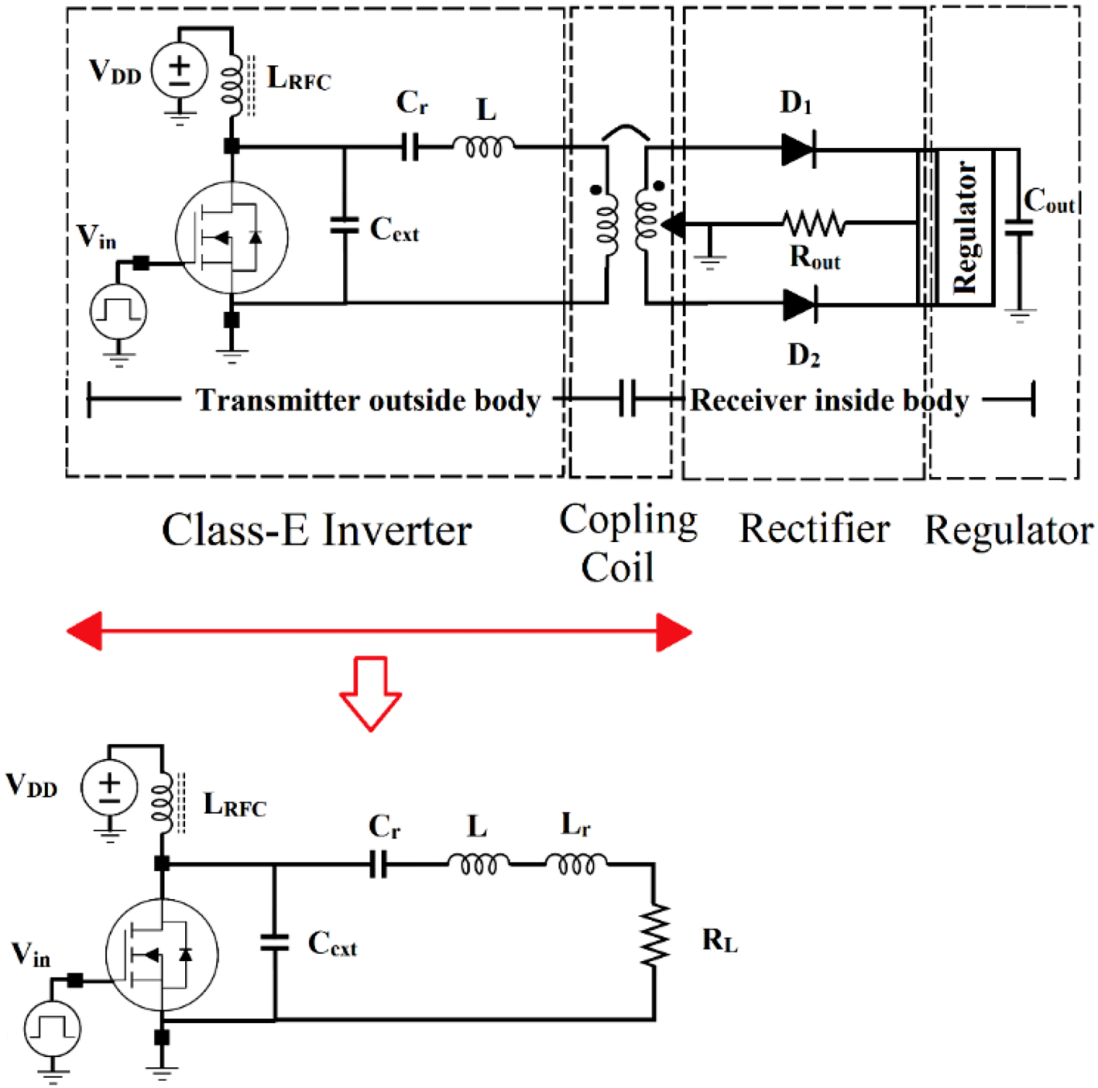
The presented circuit of a WPT is used in biomedical implants.

The proposed block diagram of a WPT used in biomedical implants is shown in Fig. 2. Due to the high efficiency of switching inverters, they are used in the wireless power supply system in the power transmitter part. In wireless implants, the transmitter induces the required power into the implant circuit with an inductive couple that transmits the power through the coils. In the receiver part of the body, a rectifier and a regulator are utilized to create the required power. The presented circuit of a WPT is shown in Fig. 3.

Class E inverter is used in many WPTs due to its simplicity of structure and high efficiency at the operating frequency. The important elements of the Class E inverter circuit are the MOSFET, which acts as a switch, a resonant circuit at the desired operating frequency, and an external capacitor. In a class E inverter, the transistor is biased in the cut-off region, with a sufficient gate driving amplitude to make it to operate alternatively between the cut-off and the linear region. Nonlinear elements of the transistor include the drain-source capacitor and the gate-drain capacitor, which have a non-linear voltage dependence. The drain-source on-resistor has a nonlinear temperature dependence. The output series resonance circuit followed by a phase difference inductor is designed to produce the output signal at the operating frequency with the lowest power loss. A choke is used between the drain of the transistor and the power supply to create low-ripple DC current and isolate ac signals from DC. In most designs, the transistor is considered an ideal switch, which is modeled as a short circuit in the ON-state and as an open circuit in the OFF-state, but in the ON-state, the transistor has a resistance that depends on the transistor parameters.

### Transistor temperature dependency

All passive and active elements have internal resistance, considering this resistance in the design process causes theoretical and fabricated responses to be in good agreement with each other. The most dependence of the transistor on the temperature is related to this resistance, which is tied to the drain-source. In switching inverters, the transistor operates in the triode region in the ON-state and operates in the cut-off region in the OFF-state. The relation of the transistor current in the triode region is defined as^41^:

1 $$ i_{T} = \mu_{n} C_{ox} \frac{W}{L}\left[ {\left( {V_{GS} - V_{th} } \right)V_{DS} - \frac{1}{2}V_{DS}^{2} { }} \right] $$

In the deep triode region, V_DS_ ≤ 2(V_GS_ − V_th_), it can be obtained:

2 $$ i_{T} \approx \mu_{n} C_{ox} \frac{W}{L}\left[ {\left( {V_{GS} - V_{th} } \right)V_{DS} { }} \right] $$

The relationship of the ON-state drain-source resistance can be presented as^41^:

3 $$ R_{ON} = \frac{1}{{\mu_{n} C_{ox} \frac{W}{L}\left[ {\left( {V_{GS} - V_{th} } \right)} \right]}} $$

where *i*_*T*_ is the transistor current, *V*_*GS*_ is the gate-source voltage, *V*_*DS*_ is the drain-source voltage, ${\mu }_{n}$ is mobility, *V*_*th*_ is the threshold voltage, and ${C}_{ox}$ is the oxide capacitor per unit area. *W* is 100u and *L* is 100u for IRF510. In (3), ${\mu }_{n}$ and *V*_*th*_ are temperature-dependent parameters that lead to the temperature dependence of ON-state drain-source resistance. Firstly, the effect of temperature variations on *V*_*th*_ is investigated. *V*_*th*_ is obtained as^42^:

4 $$ V_{th} = 2\emptyset_{F} + \frac{{\sqrt {2\varepsilon_{si} qN_{a} \left( {2\emptyset_{F} } \right)} }}{{C_{ox} }} $$

where $q$ is the electron charge and ${N}_{a}$ is the acceptor doping density, ${\varepsilon }_{si}$ is the dielectric constant of the semiconductor and $2{\mathrm{\varnothing }}_{F}$ is twice the bulk potential. Where for the P substrate, the bulk potential is obtained as follows^42^:

5 $$ \emptyset_{F} = V_{T} ln\frac{{N_{a} }}{{n_{i} }} $$

${V}_{T}$ is the thermal voltage and ${n}_{i}$ is the intrinsic carrier density. The ${V}_{T}$ and ${n}_{i}$ depend on temperature and are described as follows^42^:

6 $$ V_{T} = \frac{KT}{q} $$

7 $$ n_{i} = \sqrt {N_{C} N_{v} } e^{{ - E_{g} /2KT}} $$

*N*_*C*_ is the Effective density of states in the conduction band, *N*_*v*_ is the Effective density of states in the valence band, *E*_*g*_ is the semiconductor Energy bandgap, and *K* is Boltzmann’s constant. The energy bandgap of a semiconductor is related to temperature and is expressed as follows^42^:

8 $$ E_{g} \left( T \right) = - E_{g} \left( 0 \right) - \frac{{\alpha T^{2} }}{T + \beta } $$

By substituting (6), (7), and (8) in (5), the ${\mathrm{\varnothing }}_{F}$ is obtained by^42^:

9 $$ \emptyset_{F} = \frac{KT}{q}ln\frac{{N_{a} }}{{\sqrt {N_{C} N_{v} } e^{{ - E_{g} \left( 0 \right) - \frac{{\alpha T^{2} }}{T + \beta }/2KT}} }} $$

Mobility is the second parameter that depends on the temperature. Mobility decreases with increasing temperature with coefficient T^-2.4^ and is defined as below^42^:

10 $$ \mu_{n} = \mu_{n0} T^{ - 2.4} $$

where ${\mu }_{n0}$ varies based on the amount of silicon impurity. By substituting all the parameters in (3), the equation of resistance based on temperature is obtained as:

11 $$ R_{ON} = \frac{1}{{\mu_{n0} T^{ - 2.4} C_{ox} \frac{W}{L}\left[ {\left( {\begin{array}{*{20}c} {V_{GS} - 2\frac{KT}{q}ln\frac{{N_{a} }}{{\sqrt {N_{C} N_{v} } e^{{\left( { - E_{g} \left( 0 \right) - \frac{{\alpha T^{2} }}{T + \beta }} \right)/2KT}} }}} \\ { + \frac{{\sqrt {2\varepsilon_{si} qN_{a} 2\frac{KT}{q}ln\frac{{N_{a} }}{{\sqrt {N_{C} N_{v} } e^{{\left( { - E_{g} \left( 0 \right) - \frac{{\alpha T^{2} }}{T + \beta }} \right)/2KT}} }}} }}{{C_{ox} }}} \\ \end{array} } \right)} \right]}} $$

The constant parameters for silicon in (11) are defined as below^42^:

$$ \alpha = 0.473\frac{{{\text{meV}}}}{{\text{K}}}\quad \beta = 636{ }\,{\text{K}}\quad E_{g} \left( 0 \right) = 1.166\,{\text{ eV}} $$

Temperature changes are caused by changes in ambient temperature and junction temperature, which causes power dissipation in the transistor. It can be seen from (11), *R*_*ON*_ is a function of *V*_*th*_ and mobility, which decreases with increasing temperature.

**Figure 4:**
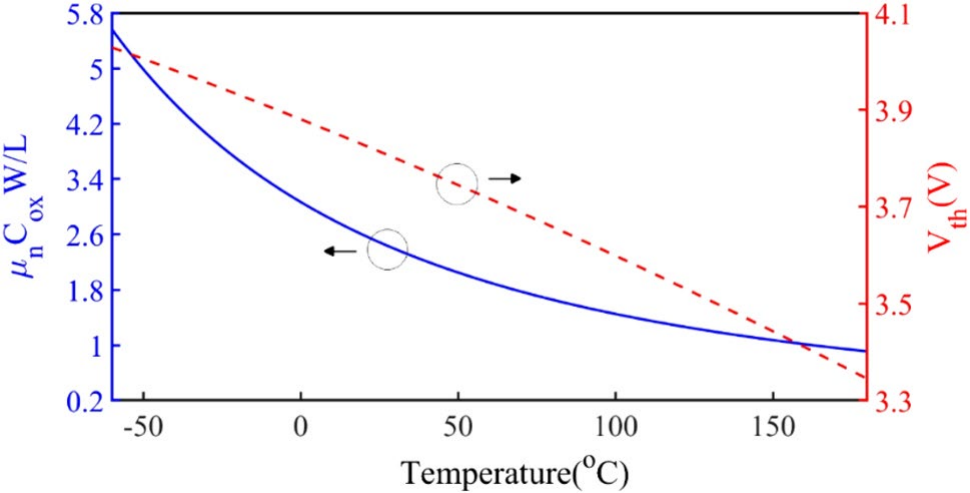
The dependence of *V*_*th*_ and mobility as a function of temperature for the IRF510 transistor.

**Figure 5:**
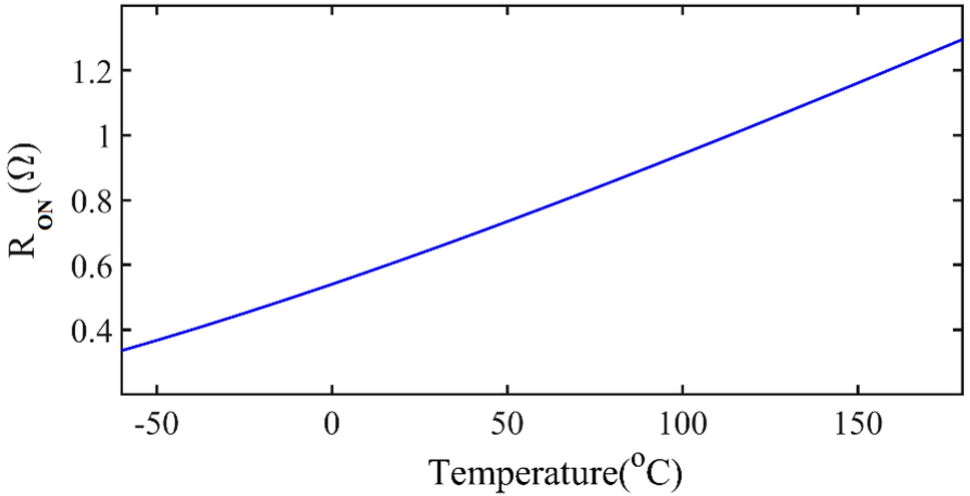
The dependence of *R*_*ON*_ as a function of temperature for the IRF510 transistor when VGS = 5.2 V.

Figure 4 shows the dependence of *V*_*th*_ and mobility as a function of temperature for IRF510 transistor. The change of R_ON_ based on temperature variations when V_GS_ = 5.2 V is shown in Fig. 5. *R*_*ON*_ has an increasing trend with increasing temperature.

### Presented analysis for the class E inverter

**Figure 6:**
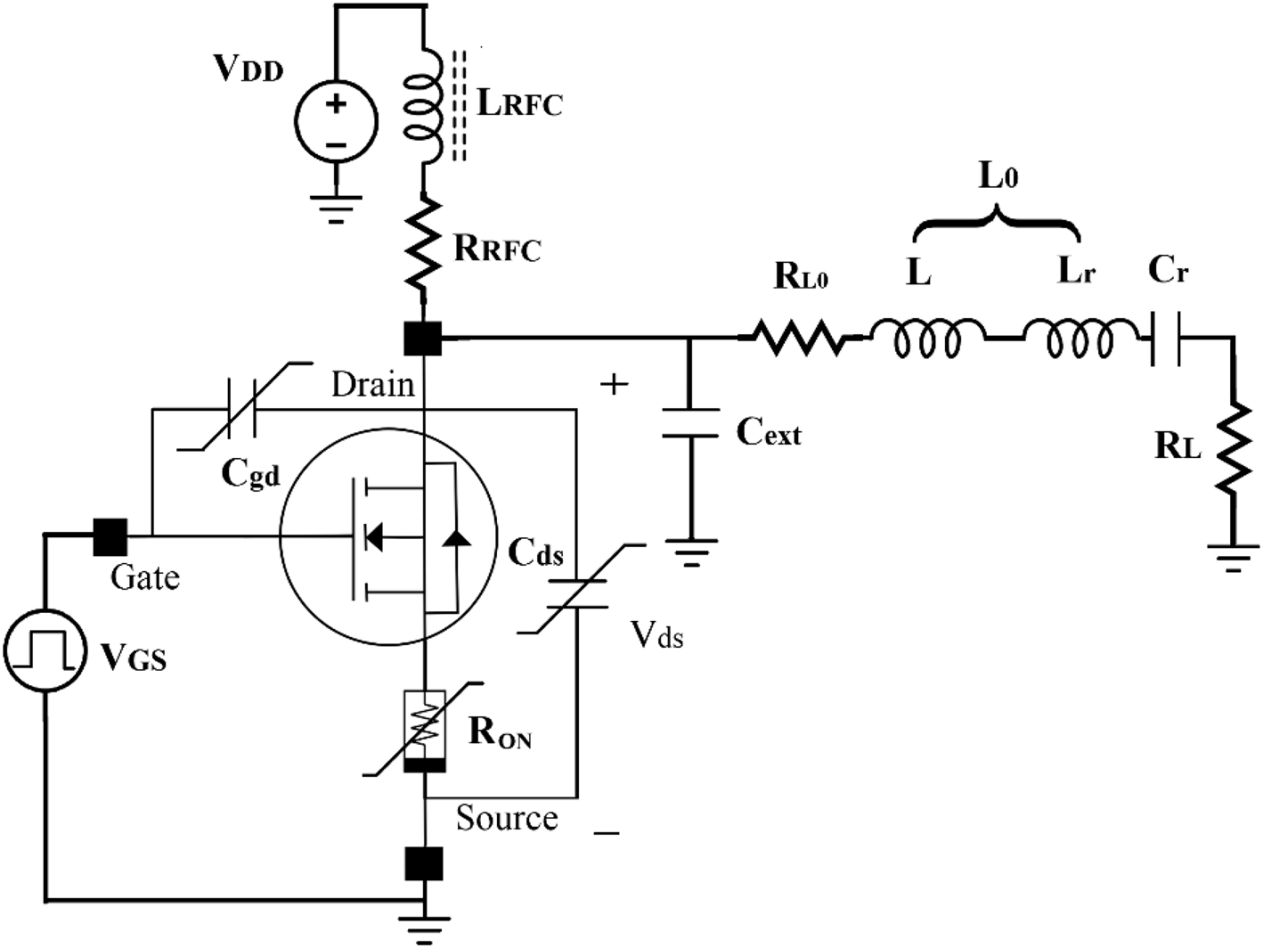
The equivalent circuit of the simple class E (SCE) inverter circuit.

Figure 6 shows the equivalent of the simple class E (SCE) inverter circuit which consists of:

- The transistor considers the nonlinear capacitances (*C*_*ds*_ and *C*_*gd*_).
- *R*_*ON*_ is the resistance of the transistor ON-state.
- *C*_*ext*_ is the fixed external capacitor.
- *L*_*RFC*_ is used as the ac blocker.
- *R*_*RFC*_ is the resistance of the *L*_*RFC*._
- *L* acts as a phase difference inductor.
- *R*_*L0*_ is the resistance of *L* + *Lr*.
- *C*_*r*_ + *L*_*r*_ just passes the first harmonic and blocks other harmonics.

**Figure 7:**
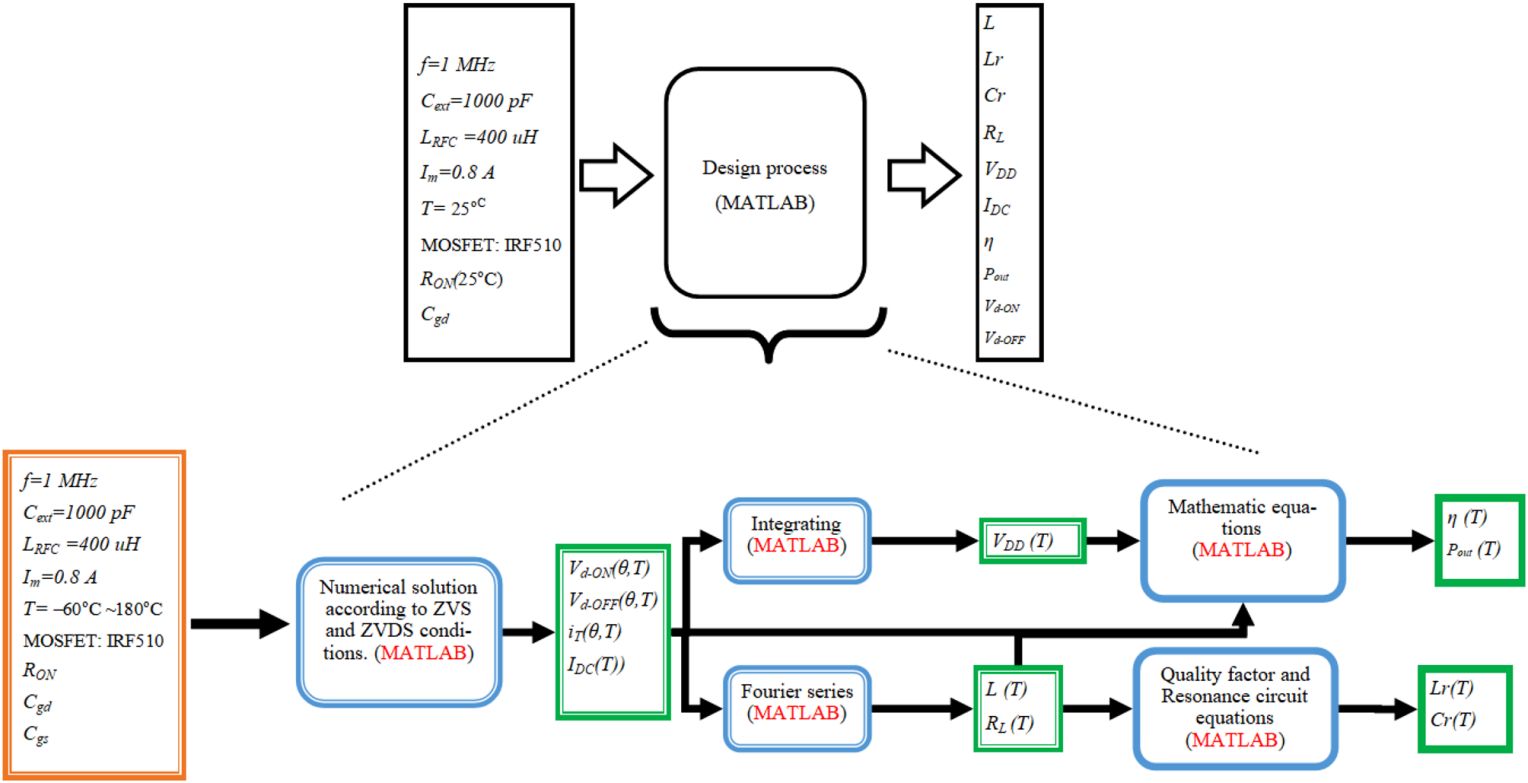
Block diagram of the Presented Analysis for the Class E Inverter.

The element values and specifications of the class E inverter in − 60 °C ~ 180 °C temperature ranges are obtained by numerical solution according to ZVS and ZVDS conditions including nonlinear *R*_*ON*_, *C*_*ds*_, and *C*_*gd*_. Figure 7 shows a block diagram of the design process and methodology that is used. In the design, the input parameters are operating frequency, peak output current *I*_*m*_, external capacitances *C*_*ext*_, and chock inductance *L*_*RFC*_, which are known. Also, the output parameters are *L*, *L*_*r*_, *C*_*r*_, *R*_*L*_, *V*_*DD*_, efficiency, output power, *V*_*d−ON*_, *V*_*d−ON* ,_ and *I*_*DC*_, which are obtained according to the temperature variation.

A square pulse with a duty cycle of 50% is applied to the gate transistor. When the transistor is OFF (0˂Ɵ < π), the gate-source voltage is zero, leaving only the nonlinear capacitors of the transistor. The following relationship can be presented:

12 $$ Transistor \,off \to - I_{DC} + \sum\nolimits_{n = 1}^{\infty } {I_{mn} \sin \left( {n\theta + \varphi_{n} } \right)} + \omega \left( {C_{ext} + C_{ds} + C_{gd} } \right)\frac{{dV_{d - OFF} }}{d\theta } = 0 $$

In (12), ${V}_{d-OFF}$ is the drain-source voltage in the OFF-state, *I*_*DC*_ is the DC current injected from *V*_*DD*_, and because of *L*_*RFC*_ operation, this current has a very little ripple. *I*_*mn*_ is the nth harmonic peak of the output current. The nonlinearity of transistor capacitors (*C*_*ds*_ and *C*_*gd*_) are considered for more accuracy in the design and their relationship is as follows^38^:

13 $$ C_{ds} = \frac{{C_{j01} }}{{\left( {1 + \frac{{V_{d - OFF} }}{{V_{{bi_{1} }} }}} \right)^{{m_{1} }} }},\,C_{gd} = \frac{{C_{j02} }}{{\left( {1 + \frac{{V_{d - OFF} }}{{V_{{bi_{2} }} }}} \right)^{{m_{2} }} }} $$

where ${V}_{d-OFF}$ is the drain-to-source transistor voltage in the OFF-state, V_bi1_ and V_bi2_ are the built-in voltage of the pn junction, C_j01_ is the drain-to-source capacitance at ${V}_{d-OFF}$, C_j02_ is the gate-to-drain capacitance at ${V}_{d-OFF}$, m_1_ is the grading coefficient for the nonlinear drain-to-source capacitance and m_2_ is the grading coefficient for the nonlinear gate-to-drain capacitance. By substituting (13) in (12) and integrating from *θ* we have:

14 $$ \begin{aligned} & \frac{{\omega C_{j01} V_{bi1} }}{{1 - m_{1} }}\left[ {\left( {1 + \frac{{V_{d - OFF} }}{{V_{bi1} }}} \right)^{{1 - m_{1} }} - 1} \right] + \frac{{\omega C_{j02} V_{bi2} }}{{1 - m_{2} }}\left[ {\left( {1 + \frac{{V_{d - OFF} }}{{V_{bi2} }}} \right)^{1 - m2} - 1} \right] + \omega C_{ext} V_{d - OFF} \\ & \quad = \, I_{DC} \theta + \mathop \sum \limits_{n = 1}^{\infty } \frac{{I_{mn} }}{n}\left[ {\cos (n\theta + \varphi_{n} ) - \cos (\varphi_{n} )} \right] \\ \end{aligned} $$

The parameters of IRF510 MOSFET are listed in Table 1^43^.

**Table 1:** Parameters of the IRF510 MOSFET.

Parameters	*C*_*j01*_ (pF)	*V*_*bi*1_ (V)	*m*_1_	*C*_*j02*_ (pF)	*V*_*bi*2_ (V)
Value	298	0.774	0.423	185	0.5

Parameters	*m*_2_	*V*_*th*_ (V)	*μ*_*n*_*C*_*ox*_	*W* (μm)	*L* (μm)
Value	0.651	3.82703	2.48457	100	100

When the transistor is ON (π˂Ɵ < 2π), the gate-source voltage is high, R_ON_ impact is added to the structure. *i*_*T*_ is the current passing through the transistor. This current is considered zero when the transistor is in OFF-state due to the high resistance of the transistor. By utilizing the current division in the drain node, the below equation can be obtained:

15 $$ i_{T} = I_{DC} - \sum\nolimits_{n = 1}^{\infty } {I_{mn} \sin \left( {n\theta + \varphi_{n} } \right)} \times \frac{{Z_{{C_{ds} }} II Z_{{C_{gd} }} IIZ_{{C_{ext} }} }}{{Z_{{C_{ds} }} IIZ_{{C_{gd} }} IIZ_{{C_{ext} }} + R_{ON} }} $$

where ‘Z’ and ‘II’ mean impedance and parallel, respectively.

The ZVS and ZVDS conditions are applied to the transistor voltage to avoid overlap between voltage and current at π. These conditions create minimal consumption in the design. Applying these conditions to Eqs. (12) and (15):

16 $$ ZVDS \to \frac{{dV_{d - OFF} \left( \pi \right)}}{d\theta } = 0 \to - I_{DC} + \sum\nolimits_{n = 1}^{\infty } {\left( { - 1} \right)^{n} I_{mn} \sin \left( {\varphi_{n} } \right)} = 0 $$

17 $$ ZVS = 0 \to V_{d - OFF} \left( \pi \right) = 0 \to I_{DC} \pi + \sum\nolimits_{n = 1}^{\infty } {\frac{{I_{mn} }}{n}\left[ {\left( { - 1} \right)^{n} - 1} \right]\cos (\varphi_{n} ) = 0} $$

**Figure 8:**
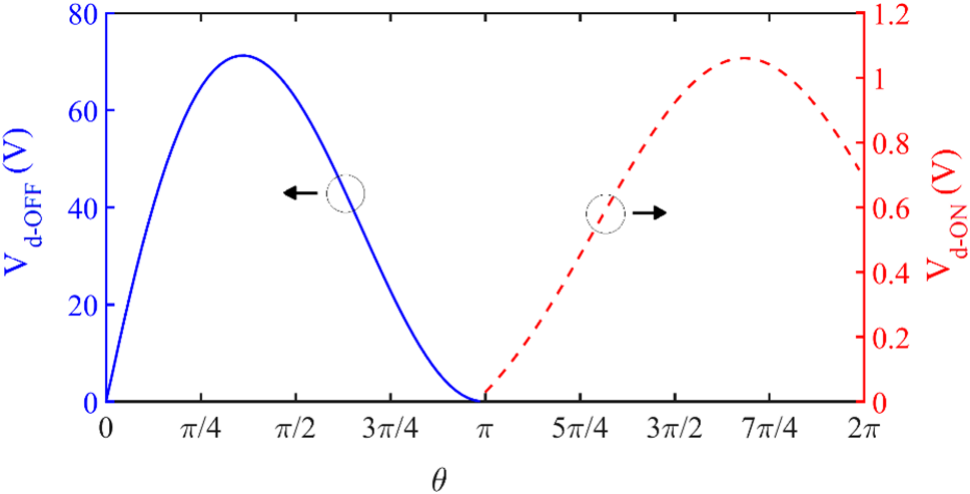
V_d_ in on and OFF-states versus Ɵ.

The output resonance circuit of the transistor is designed with high-quality factor, so only the first harmonic is considered in the design and the rest of the harmonics are ignored. Therefore, according to (16) and (17), the amount of phase difference required to meet the conditions of ZVS and ZVDS is equal to ${\varphi }_{n}=$ − 0.567 rad. Figure 8 shows the drain voltage at 25 °C as well as determines the drain voltage peak value in the ON and OFF states. It can be seen that the drain voltage in the ON-state has a significant value, but it has been neglected in previous class E inverter designs.

The drain voltage in the ON-state according to *R*_*ON*_ can be obtained as below:

18 $$ V_{d - ON} = i_{T} \times R_{ON} $$

where ${i}_{T}$ is the current of the transistor in ON-state, which is presented in (15).

The voltage of the DC power supply according to the drain voltage can be obtained. 0 to π indicates the OFF-state, and π to 2π indicates the ON-state of the transistor, V_DD_ can be calculated as follows:

19 $$ V_{DD} = \frac{1}{2\pi }\left[ {\int_{0}^{\pi } {V_{d - OFF} \left( \theta \right)d\theta } + \int_{\pi }^{2\pi } {V_{d - ON} \left( \theta \right)d\theta } } \right] + R_{RFC} I_{DC} $$

**Figure 9:**
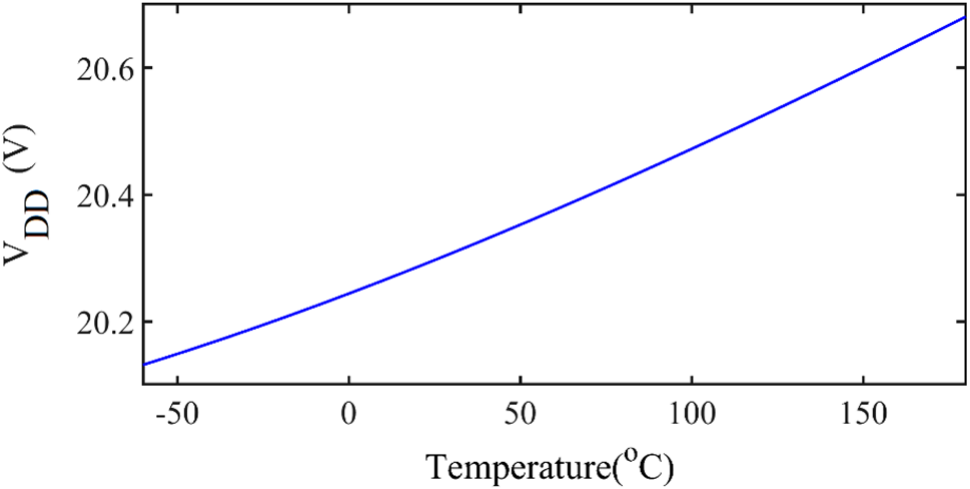
The value of *V*_*DD*_ based on temperature.

To have a constant output current with ZVS and ZVDS conditions versus temperature changes, the circuit parameters like *V*_*DD*_, *L*_*r*_, *C*_*r*_, *L*, and *R*_*L*_ must be changed. According to (19), *V*_*DD*_ versus temperature changes is obtained and shown in Fig. 9.

**Figure 10:**
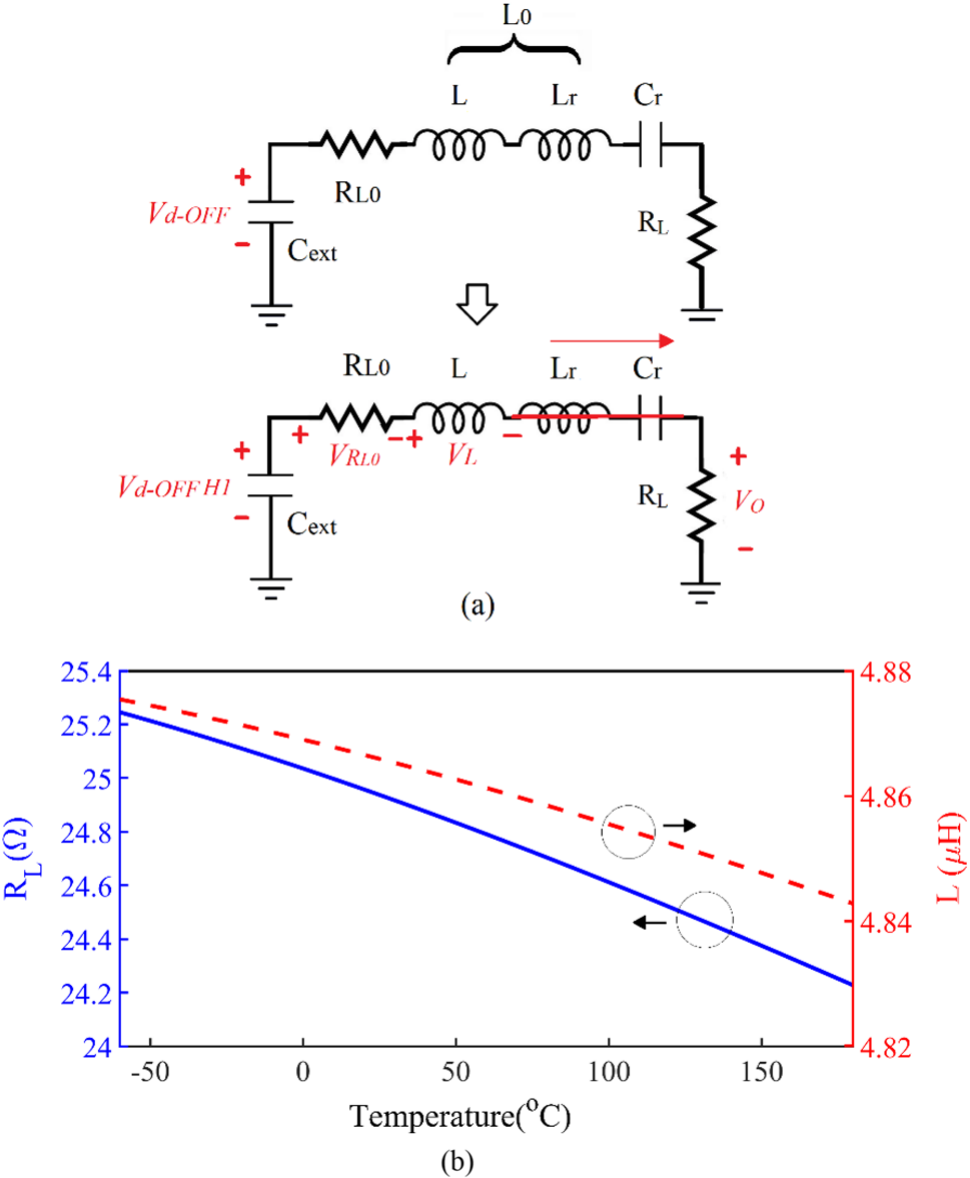
(**a**) The output resonant circuit in the main harmonic. (**b**) The *R*_*L*_ and *L* for establishing ZVS and ZVDS.

As temperature increases, *V*_*DD*_ should be increased to compensate for the drop in drain voltage in the OFF-state. Figure 10a shows the output resonant circuit. At the fundamental harmonic, which is considered to be 1 MHz, the resonant circuit is simplified, as shown in Fig. 10a. By employing KVL in the circuit:

20 $$ - V_{d - OFF} H1 + V_{{R_{L0} }} + V_{L} + V_{o} = 0 $$

In (20), ${V}_{d-OFF}H1$ is the first harmonic of* V*_*d−OFF*_, *V*_*L*_ is the phase difference inductor voltage, *V*_*O*_ is the output voltage, and ${V}_{{R}_{L0}}$ is the *R*_*L0*_ voltage. By substituting the first harmonic of the out current in (20):

21 $$ - V_{d - OFF} H1 + R_{L0} \times I_{m1} {\text{sin}}\left( {\theta + \varphi_{1} } \right) + 2\pi f \times L \times I_{m1} {\text{cos}}\left( {\theta + \varphi_{1} } \right) + R_{L} \times I_{m1} {\text{sin}}\left( {\theta + \varphi_{1} } \right) = 0 $$

By solving (21) we have:

22 $$ \left| {V_{d - OFF} H1} \right| = I_{m1} \sqrt {\left( {2\pi f \times L} \right)^{2} + \left( {R_{L} + R_{L0} } \right)^{2} } $$

23 $$ \varphi_{{V_{d - OFF} H1}} = \varphi_{1} + tan^{ - 1} \left( {\frac{2\pi f \times L}{{R_{L} + R_{L0} }}} \right) $$

where $\left|{V}_{d-OFF}H1\right|$ and ${\varphi }_{{V}_{d-OFF}H1}$ are the absolute value and phase of the fundamental harmonic of* V*_*d−OFF*_, respectively. By solving (22) and (23) the value of output resistance and phase difference inductor are calculated as follows:

24 $$ R_{L} = \sqrt {\frac{{\left| {V_{d - OFF} H1} \right|^{2} }}{{I_{m1}^{2} \left( {1 + tan^{2} \left( {\varphi_{{V_{d - OFF} H1}} - \varphi_{1} } \right)} \right)}}} - R_{L0} $$

25 $$ L = \frac{1}{2\pi f}\sqrt {\frac{{\left| {V_{d - OFF} H1} \right|^{2} \times tan^{2} \left( {\varphi_{{V_{d - OFF} H1}} - \varphi_{1} } \right)}}{{I_{m1}^{2} \left( {1 + tan^{2} \left( {\varphi_{{V_{d - OFF} H1}} - \varphi_{1} } \right)} \right)}}} $$

By solving (24) and (25), the optimal load resistance values (*R*_*L*_) and *L* that are suitable for creating ZVS and ZVDS conditions at different temperatures are calculated. The calculated *R*_*L*_ and *L* are shown in Fig. 10b. As the temperature increases, the value of these parameters should be reduced to achieve the conditions of ZVS and ZVDS and have a constant output current.

**Figure 11:**
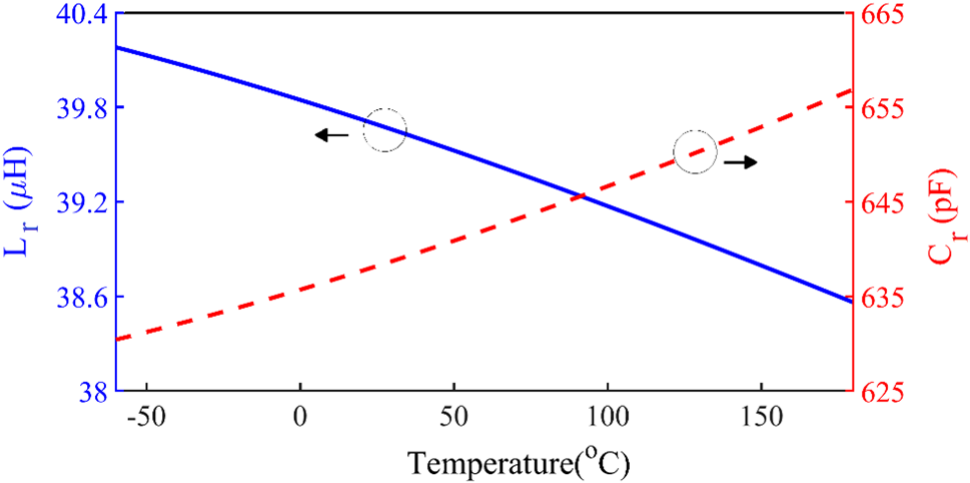
The L_r_ and C_r_ of the series resonance.

In this design, the quality factor in the resonant circuit is considered to be 10, so that the output current waveform is close to a pure sinusoid. According to (26) and (27), the value of the inductor and capacitor of the series resonant are obtained based on the temperature, as shown in Fig. 11. As the temperature increases, to access the ZVS and ZVDS conditions and constant output current, the inductor should be decreased and the capacitor should be increased.

26 $$ Q = \frac{{2\pi fL_{r} }}{{R_{L} }} \to L_{r} = \frac{{Q \times R_{L} }}{2\pi f} $$

27 $$ f = \frac{1}{{2\pi \sqrt {L_{r} \times C_{r} } }} \to C_{r} = \frac{1}{{\left( {2\pi f} \right)^{2} L_{r} }} $$

In a real Class E inverter, the efficiency is not 100% due to the presence of parasitic elements. The efficiency can be calculated by dividing the power delivered to the load over the total power consumption in the circuit as follows:

28 $$ \eta = \frac{{P_{O} }}{{P_{O} + P_{loss} }} $$

The total power consumption of the circuit is expressed as:

29 $$ P_{loss} = P_{{R_{RFC} }} + P_{{R_{LO} }} + P_{{R_{on} }} $$

where ${P}_{{R}_{RFC}}$,${P}_{{R}_{L0}}$, and ${P}_{{R}_{on}}$ are the dissipated power in *R*_*RFC*_, *R*_*L0*_, and *R*_*ON*_, respectively. The efficiency is calculated as:

30 $$ \eta = \frac{{\frac{1}{2}R_{L} I_{m}^{2} }}{{\frac{1}{2}R_{L} I_{m1}^{2} + R_{RFC} I_{DC}^{2} + 0.5R_{L0} I_{m1}^{2} + \frac{1}{2\pi }\mathop \smallint \nolimits_{0}^{2\pi } R_{ON} I_{m1} \sin \left( {\theta + \varphi_{1} } \right)d\theta }} $$

**Figure 12:**
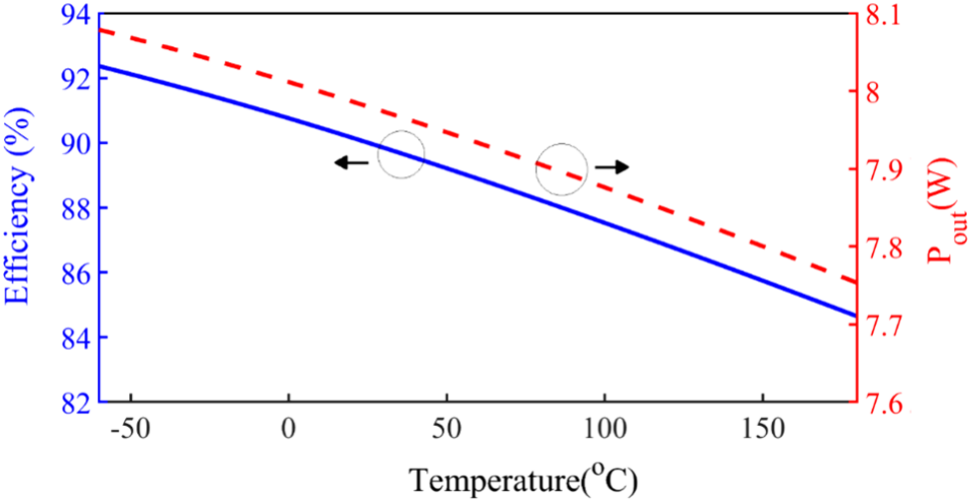
The output power and efficiency.

In order to have a constant output current and maintain the nominal conditions, the values of the circuit elements change according to the ambient and junction temperature. Figure 12 shows, that the efficiency and output power versus temperature variations when the values of the circuit elements change. As can be seen, the output power and the efficiency in the presented SCE inverter decrease with rising temperature.

### Presented design for the class E inverter at 25 °C

**Figure 13:**
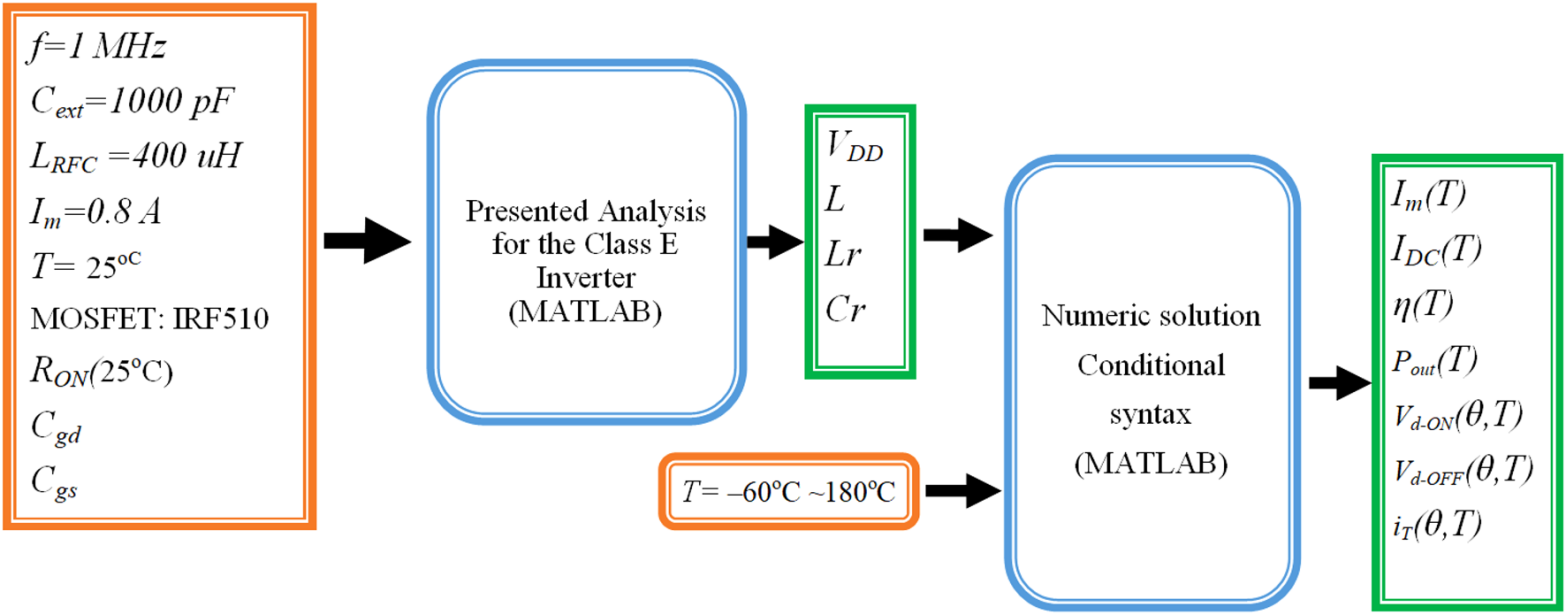
Block diagram of the Presented Design for the Class E Inverter at 25 °C.

In this section, a class E inverter at 25 °C is designed and the elements of the circuit are obtained according to previous analysis as listed in Table 2, then the effect of temperature variations on the specifications are investigated as shown by a block diagram in Fig. 13.

**Table 2:** The element’s value of the presented class E inverter at 25 °C.

Element	Values
*L* (μH) + *L*_*r*_(μH)	4.86 + 39.6
*C*_*r*_ (pF)	638.7
*R*_*L*_ (Ω)	24.9
*V*_*DD*_ (V)	20.31
*L*_*RFC*_ (μH)	400
*R*_*RFC*_(Ω)	0.3
*C*_*ext*_ (pF)	1000

**Figure 14:**
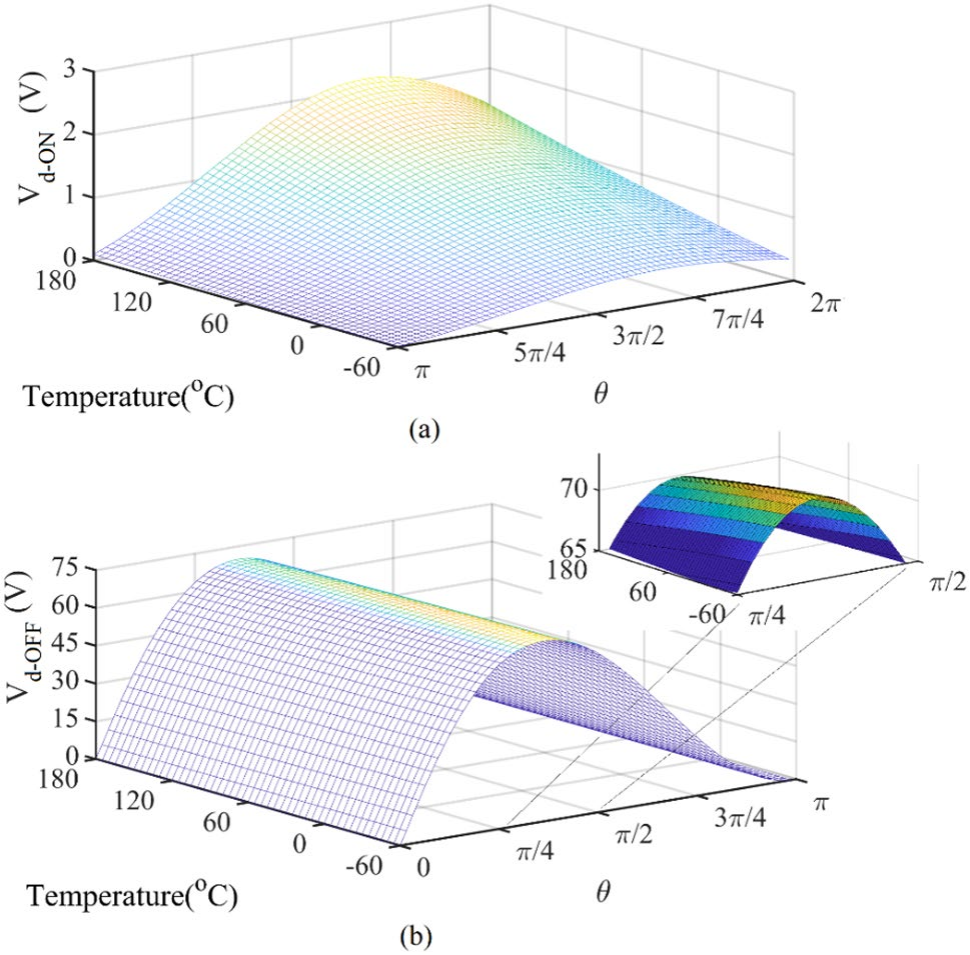
V_d_ versus temperature and Ɵ (**a**) in the ON-state (π < Ɵ < 2π), (**b**) in the OFF-state (0 < Ɵ < π).

By solving (14) and (15), the dependence of the drain voltage is calculated according to temperature and *Ɵ*, as shown in Fig. 14a,b. In the ON-state, taking into account the rising *R*_*ON,*_ the drain voltage rises, as shown in Fig. 14a. Due to the constant *V*_*DD*_, as the temperature increases, the total area under the drain voltage curve from 0 to 2π should also remain constant. Therefore, to compensate for the increase in the drain voltage in the ON state, the peak value of drain voltage in the OFF-state is reduced as shown in Fig. 14b. The drain current is considered zero in the range of 0 to *π* and the transistor resistance is assumed infinite. One of the important factors in the design is to consider the maximum voltage that can be tolerated for the transistor according to its model. If the drain voltage changes caused by temperature fluctuations are not controlled, they can damage the transistor.

**Figure 15:**
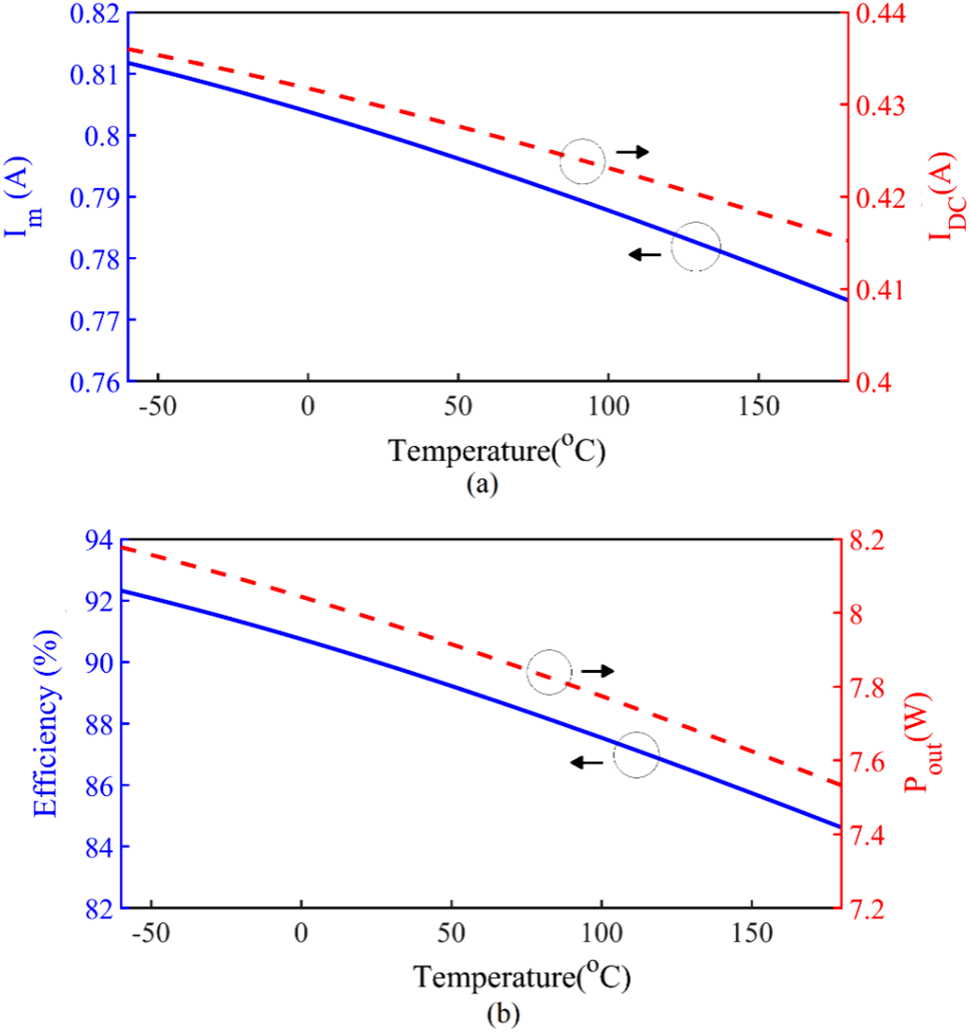
(**a**) *I*_*m*_ and *I*_*DC*_ versus temperature. (**b**) Efficiency and *P*_*out*_ versus temperature.

A change in temperature causes a change in resistance, and then the amount of output current and efficiency and output power change. Figure 15a,b show the reduction of these parameters based on temperature increment. As it is clear, these changes are significant and should be considered in the design.

### Design of the presented thermal compensation unit

**Figure 16:**
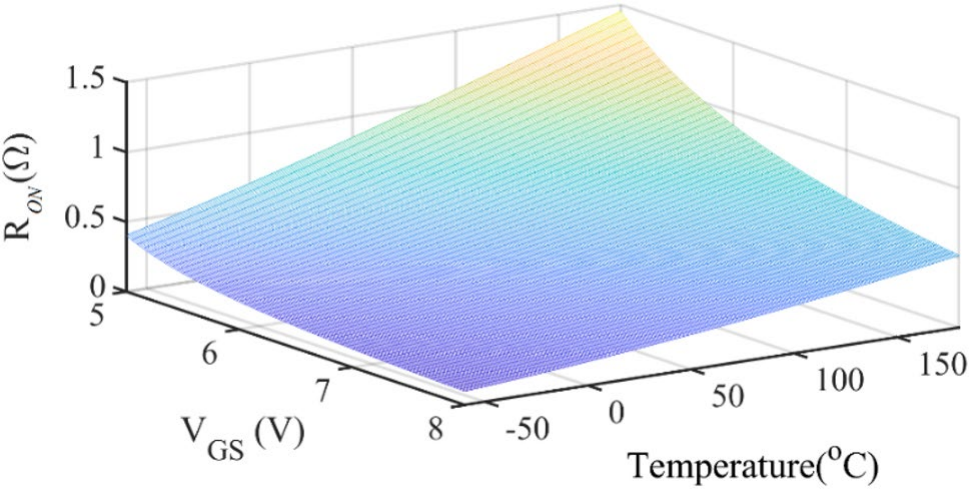
R_ON_ versus VGS and temperature.

In this section, a thermal compensation unit (TCU) is provided for the SCE. According to (11), *R*_*ON*_ has inversely related to the gate-source voltage applied to the transistor. *R*_*ON*_ versus V_GS_ and temperature is shown in Fig. 16.

**Figure 17:**
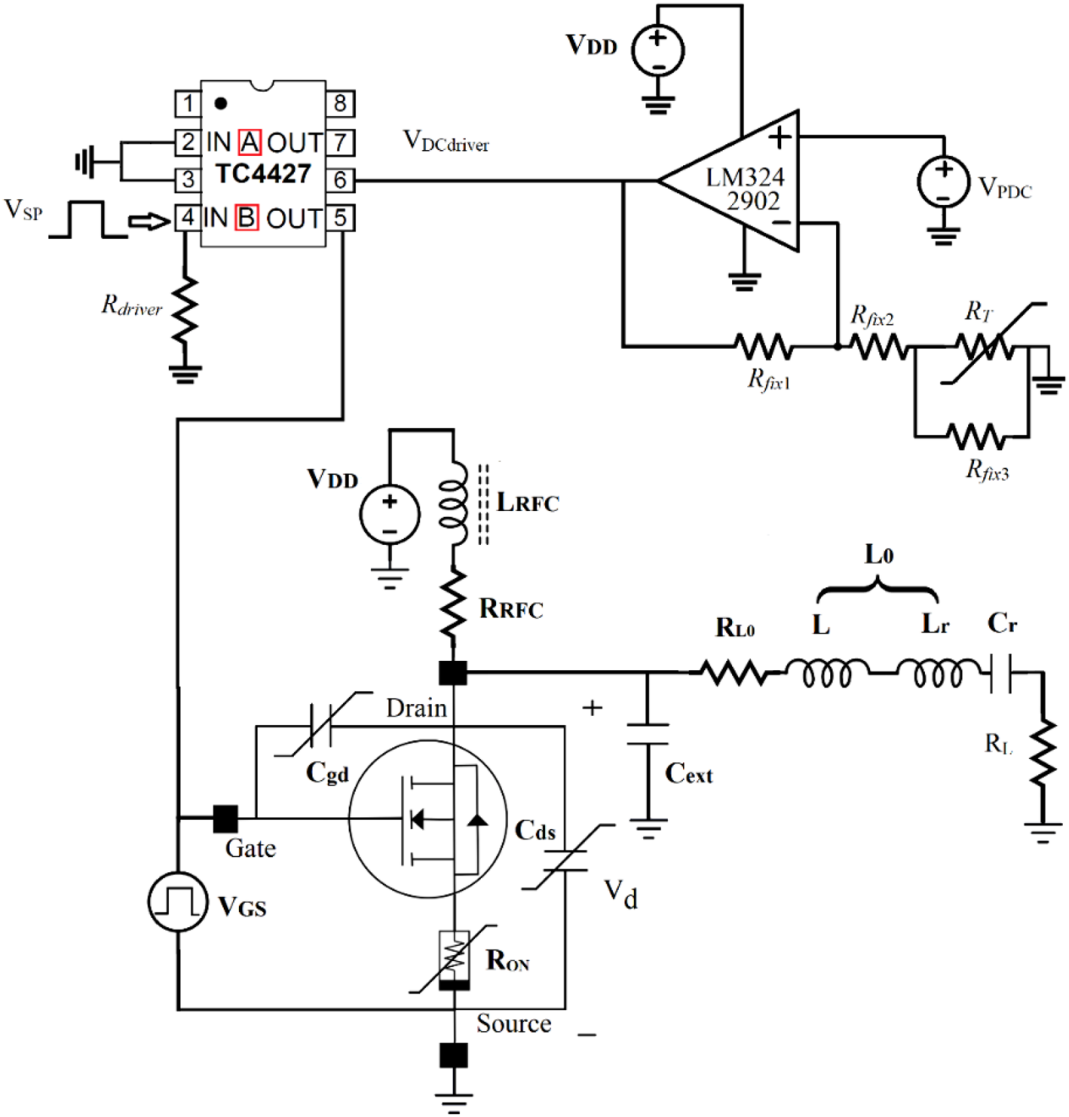
The equivalent circuit of the thermal compensated class E (TCCE) inverter.

To achieve a constant *R*_*ON*_ with temperature variations, the amount of V_GS_ in the circuit should be changed. To generate V_GS_ according to temperature changes, a compensation circuit is designed, as shown in Fig. 17.

The TCU includes:

- An OP-AMP is formed in a non-inverter state.
- Three constant resistors (*R*_*fix*1_, *R*_*fix*2,_ *R*_*fix*3_).
- *R*_*T*_ is a 10KΩ negative temperature control (NTC) resistor with 3435 part-number.
- *V*_*PDC*_ has applied voltage to the op-amp.
- TC4427 is used as the driver.

**Figure 18:**
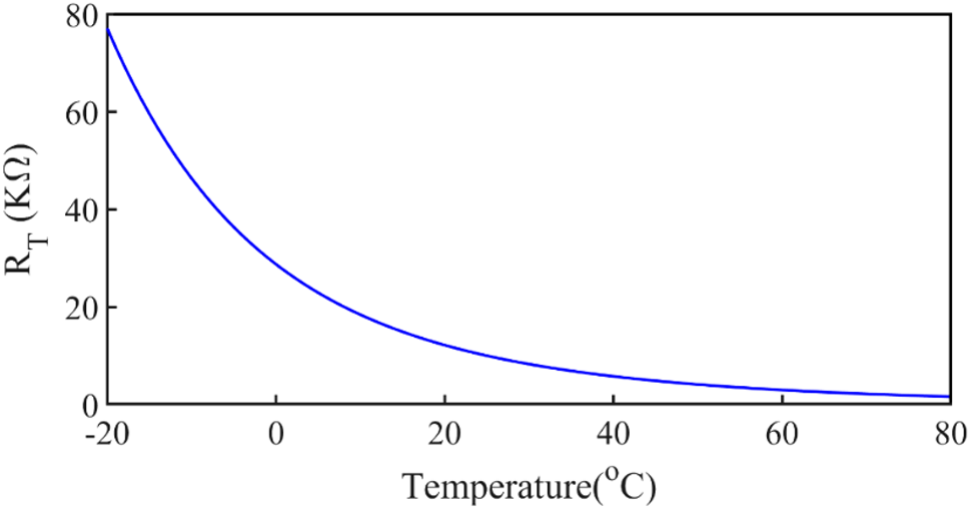
R_T_ versus temperature.

*R*_*T*_ has a nonlinear decreasing behavior with increasing temperature. The model of this resistor is 3435. The resistance value versus temperature is shown in Fig. 18.

**Figure 19:**
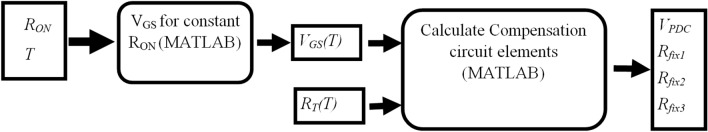
Block diagram of the TCU design flow.

**Figure 20:**
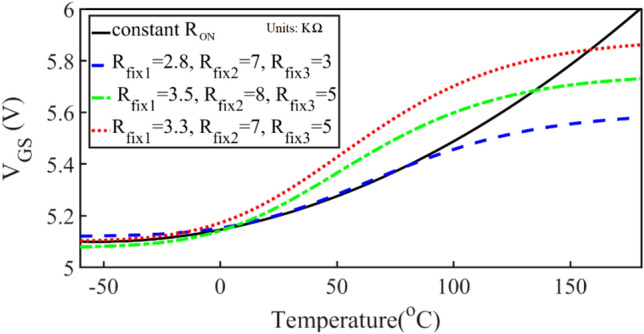
*V*_*GS*_ versus temperature for different values of R_fix1_, R_fix2_, and R_fix3_ and the constant resistance at 25 °C.

The block diagram of the TCU design process is shown in Fig. 19. The values of V_GS_ versus temperature that creates constant R_ON_ are obtained from Fig. 16 in MATLAB and plotted in Fig. 20 with a continuous-black-line. The elements value of the compensation circuit as shown in Fig. 17 are calculated in MATLAB with respect to V_GS_ and R_T_ (3435 NTC resistor) values. Figure 20 shows the created V_GS_ versus temperature with different values of *R*_*fix1*_, *R*_*fix2*_, and *R*_*fix3*_.

*V*_*DC*driver_ determines the value of the pulse signal applied to the gate and is calculated from the following equation:

31 $$ V_{DCdriver} = V_{PDC} \left( {1 + \frac{{R_{fix1} }}{{R_{fix2} + R_{T} ||R_{fix3} }}} \right) $$

**Figure 21:**
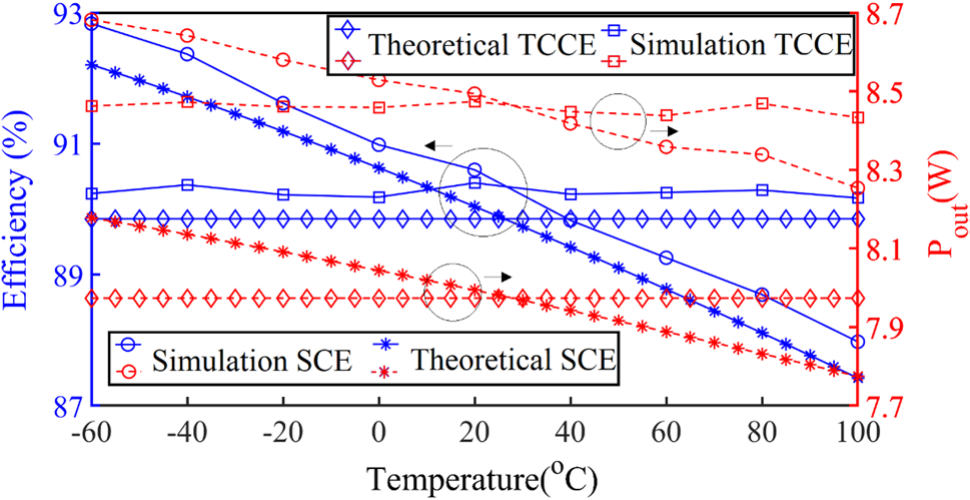
The changes in efficiency and output power of the SCE and TCCE.

Figure 20 shows, V_GS_ versus temperature for constant *R*_*ON*_ in a continuous black line. The elements of the compensation circuit, which are *R*_*fix*1_, *R*_*fix*2_, *R*_*fix*3_, and *V*_*PDC*_ should be adjusted so that the compensation circuit produces this pattern for *V*_*GS*_. The blue dashed lines with *R*_*fix*1_ = 2.8, *R*_*fix*2_ = 7, and *R*_*fix*3_ = 3 KΩ provide the best agreement between − 60 and 100 °C. The SCE is investigated with and without the compensation circuit. When the TCU is in the design, it prevents the effect of temperature variations on the inverter parameters. The changes in efficiency and output power based on the temperature in the SCE and the TCCE inverter are shown in Fig. 21, simultaneously. The compensator enables significant improvements by maintaining the simulation power and efficiency almost constant (8.46 ± 0.14 W and 90.4 ± 0.2%) within the temperature range of − 60 to 100 °C. From the comparison of efficiency and output power in the TCCE inverter and the SCE inverter, it is becoming clear that the TCU provides constant output power and efficiency versus temperature variations, which is very desirable for telecommunication circuits.

## Performance analysis result

**Figure 22:**
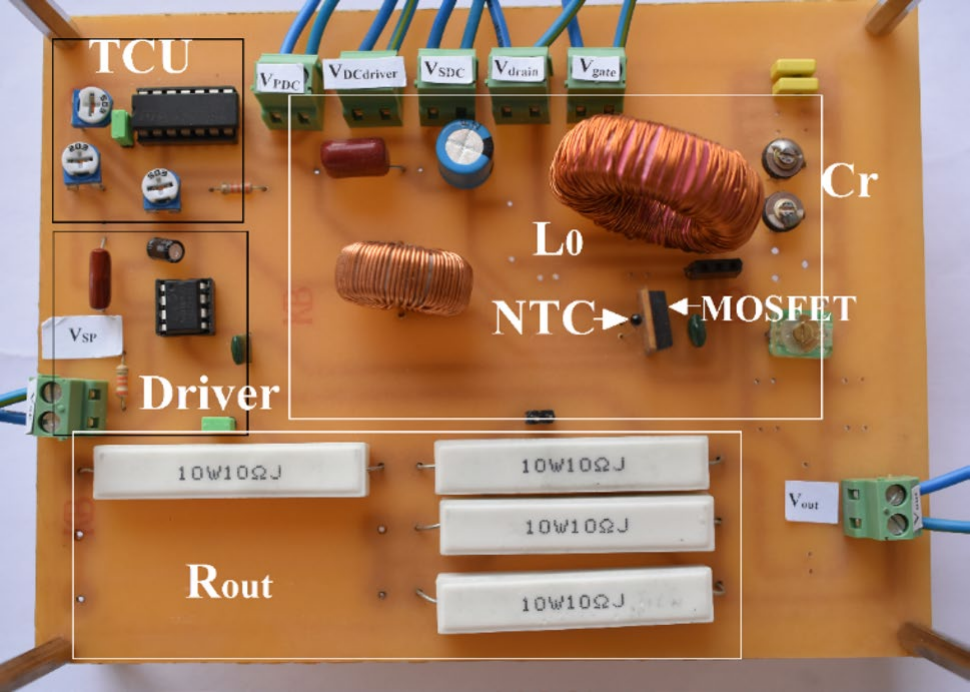
The fabricated inverter.

The presented TCCE inverter is fabricated and shown in Fig. 22. The input signal has a duty ratio of 0.5. The operation frequency is 1 MHz. As can be seen, the negative temperature control resistor is placed next to the IRF510 to sense the ambient and junction temperature simultaneously. The TCU can be isolated from the SCE inverter with a jumper so that it can be tested with and without the compensation circuit. The load resistance is made by ceramic resistors with a tolerance of 10 watts. All capacitors used are able to withstand high power.

### Comparison of theoretical, simulation, and experimental results

**Figure 23:**
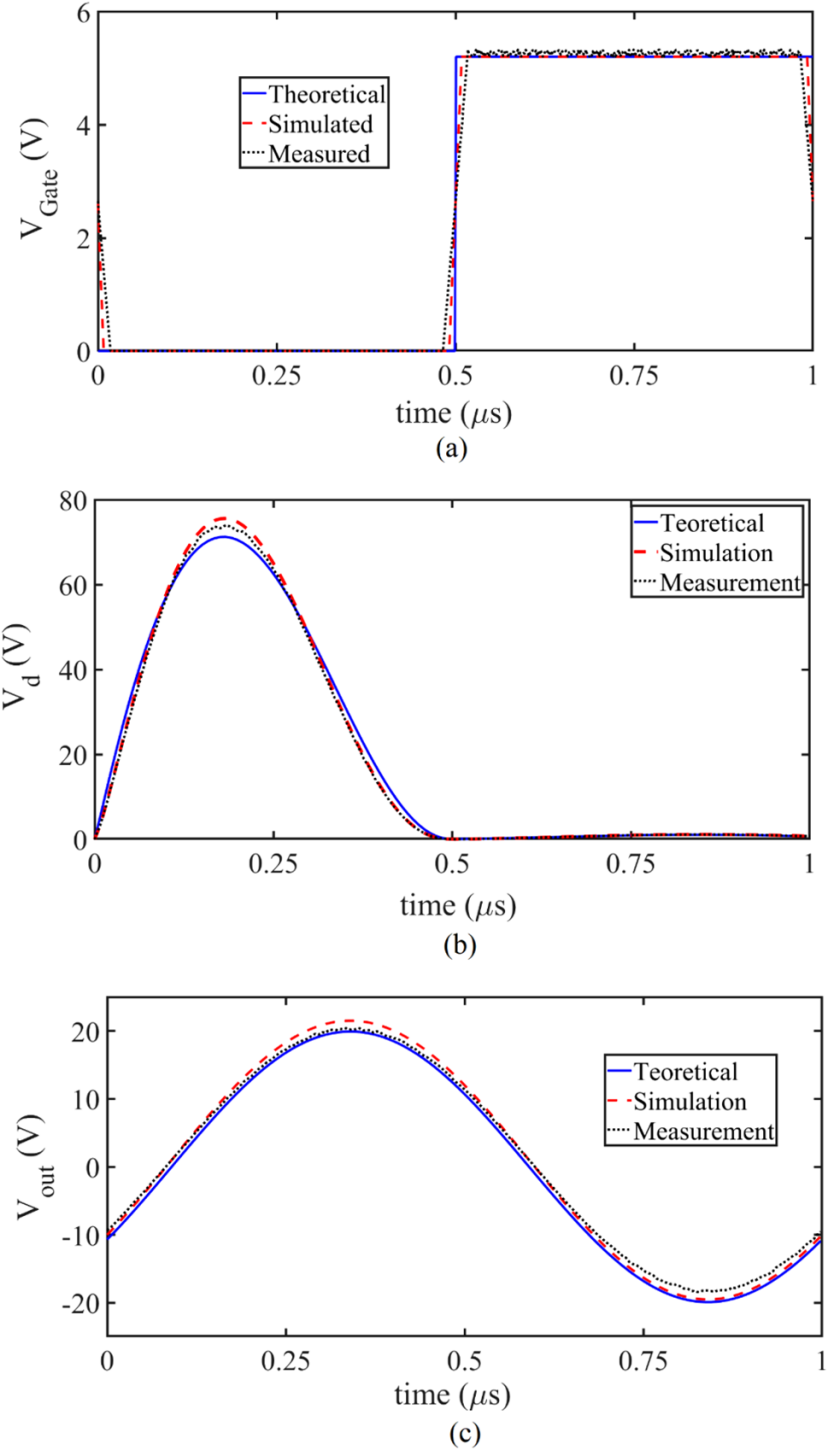
(**a**) The output driver voltage is applied to the transistor gate. (**b**) *V*_*d*_ and (**c**) the output voltage for the simulation, theoretical, and fabrication at 25 °C.

Table 3 shows the values of the elements used in the TCCE inverter. Table 4 shows the theoretical, simulated, and measured results of the presented TCCE inverter at 25 °C. As can be seen, the simulation and measurement results are very close to the theoretical results. This precision in the results is due to the consideration of all the parasitic elements of the transistor as well as the resistance of the transistor in the ON-state. Figure 23a shows the output driver voltage applied to the gate of the transistor. The value of V_GS_ is 5.2 V. The driver output capacitor causes a slow slope in the measured V_GS_. In Fig. 23b, the drain voltage for simulation, theoretical and fabrication results are shown. The output current is considered pure sinusoidal in the theoretical analysis, but in the measurement and simulation results, the output current has more harmonics, which increases the drain voltage level for simulation and measurement.

**Table 3:** The elements value of the inverter and compensation circuit.

Element	Theoretical	Simulated	Measured
*R*_*fix*1_ (kΩ)	2.8	2.8	2.8
*R*_*fix*2_ (kΩ)	7	7	7
*R*_*fix*3_ (kΩ)	3	3	3
*R*_*T*_ (kΩ) (3435)	10	10	10
*L*_*RFC*_ (μH)	400	400	400
*R*_*RFC*_(Ω)	0.3	0.3	0.3
*C*_*ext*_ (pF)	1000	1000	1000
*R*_*L0*_ (Ω)	1.2	1.2	1.5
*R*_*driver*_ (KΩ)	33	33	33
*C*_*driver*_ (pF)	500	500	500
*L* (μH) + *L*_*r*_(μH)	4.86 + 39.6	4.86 + 39.6	45
*C*_*r*_ (pF)	638.7	638.7	639
*R*_*L*_ (Ω)	24.9	24.9	25

**Table 4:** The important parameters of the inverter at 25 °C.

Parameter	Theoretical	Simulated	Measured
*V*_*DD*_ (V)	20.31	20.31	20.3
*I*_*DC*_ (A)	0.4297	0.461	0.407
*I*_*m*1_ (A)	0.8	0.824	0.77
*V*_*PDC*_ (V)	4	4	4
*P*_*out*_ (W)	7.97	8.46	7.42
*η* (%)	89.85	90.4	89.9

**Figure 24:**
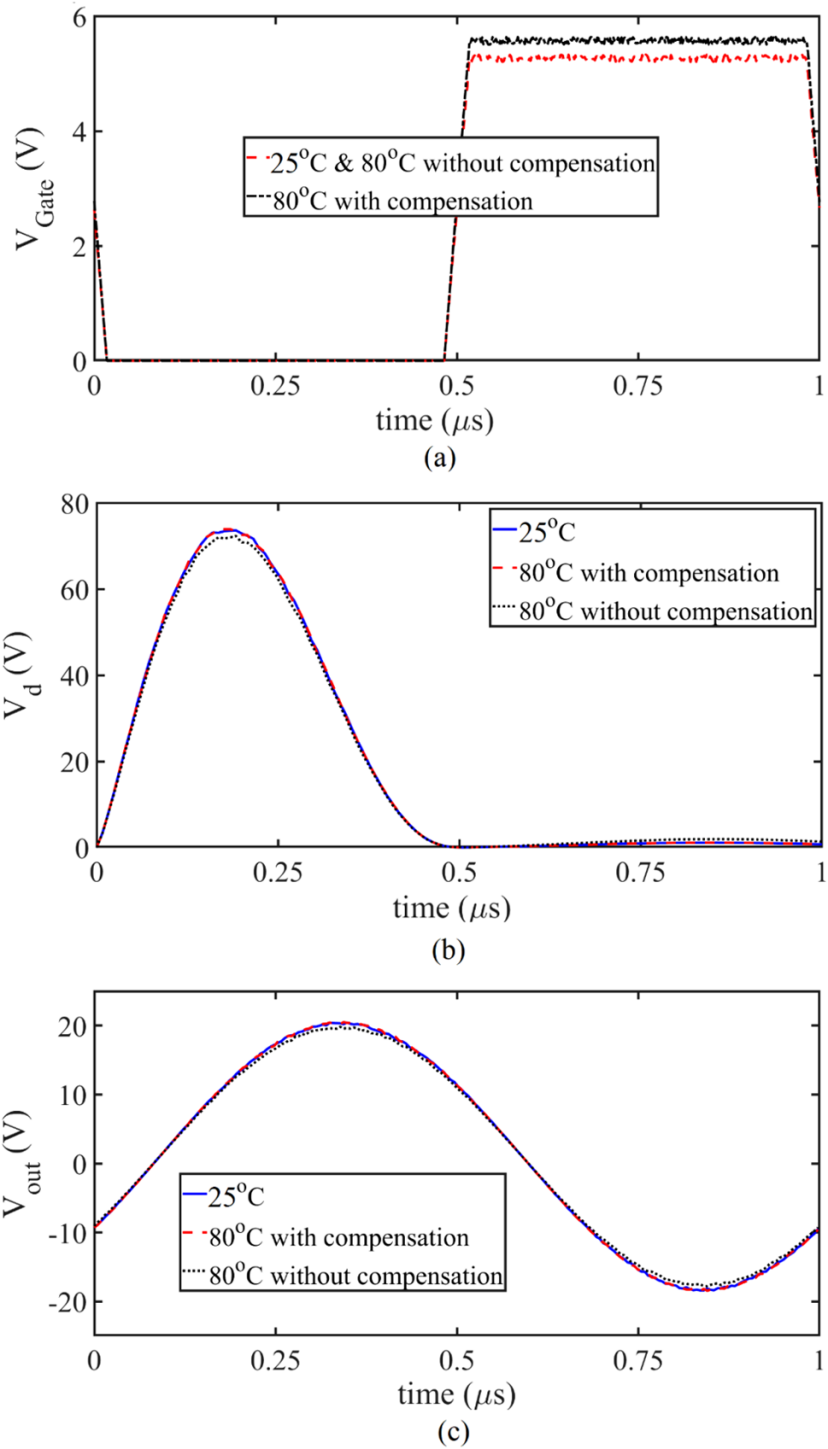
(**a**) The SCE and TCCE output driver voltage is applied to the transistor gate. (**b**) The SCE and TCCE waveform of *V*_*d*_ and (**c**) The SCE and TCCE output voltage for the simulation, theoretical, and fabrication at 25 °C and 80 °C.

Figure 24c shows the output voltage. The difference between the results is due to the increase of the resistance in the resonance path in the manufacturing process and considering the output current as a pure sinusoid. Figure 24a shows the applied gate voltage for the SCE inverter at 25 °C and 80 °C and also for the TCCE inverter at 80 °C. At a temperature of 80 °C, the value of gate voltage increases due to the operation of the compensating circuit and prevents changes in the drain voltage. As can be seen in Fig. 24b, the drain voltage in the SCE inverter at 25 °C is almost the same as the drain voltage in the TCCE inverter at 80 °C. Due to the operation of the TCU, the peak of the drain voltage remains constant. Figure 24c shows that at 80 °C in the SCE inverter, the output voltage is affected by the decreased in the drain voltage. Table 5 compares the values of the parameters measured with SCE at 25 °C and 80 °C and the parameters measured with TCCE at 80 °C. In the SCE inverter at 80 °C, the output power is 2.7% lower than the SCE inverter at 25 °C. By applying the TCCE inverter, this difference reaches 0.13%, which is very favorable. In the SCE inverter, the efficiency at 25 °C is 89.9%, which decreases to 88.1% when the temperature increases to 80 °C. As the temperature increases in the TCCE inverter, the efficiency remains constant. It is concluded that the TCU has a very positive effect on the inverter parameters. Important parameters of the class E inverter for comparing the TCCE inverter with other references are presented in Table 6. The output power and efficiency of the proposed circuit are 7.42 W and 89.9%. Output power and efficiency are in conflict with each other, in some references, despite high efficiency, they have provided lower output power. *C*_*gd*_ and *C*_*ds*_ have a non-linear relationship with the drain-source voltage and their consideration confirms the theoretical answer. None of the references consider these capacitances as nonlinear elements in design process, nor do all references consider the nonlinear effects of *R*_*ON*_.

**Table 5:** The values of the measured parameters in the state with and without compensation circuit at 80 °C.

Parameter	Measured				
Temperature (°C)	25	80			
Compensation	SCE	TCCE		SCE	
	Value	Value	Difference %	Value	Difference %
*V*_*DD*_ (V)	20.3	20.3	0	20.3	0
*I*_*DC*_ (A)	0.407	0.407	0	0.404	− 0.74
*I*_*m*1_ (A)	0.77	0.771	+ 0.13	0.759	− 1.43
*P*_*out*_ (W)	7.42	7.43	+ 0.13	7.22	− 2.7
*η* (%)	89.9	89.9	0	88.1	− 2

**Table 6:** The comparison between the presented inverter and other works.

Ref	MOSFET	*F* (MHz)	*P*_*out*_(w)	Efficiency	*V*_*GS*_	*V*_*DD*_	*C*_*gd*_	*C*_*ds*_	*R*_*ON*_	TC*
^2^	IPP530N15N3G	1	10	37.7–89.3	10	10	–	Constant	–	NO
^3^	IRFR120Z	1	4.76	93.2	NA	19.5	–	Constant	–	NO
^8^	RQ6E045BN	0.8	1.02	92	5	4.5	–	Constant	–	NO
^9^	FQT13n06	0.8	0.96	89	6	4.5	–	Constant	–	NO
^12^	–	1800	17	83	4.4	28	–	Constant	–	NO
^14^	–	1370	9.5	90.2	8.8	28	–	Constant	–	NO
^19^	0.18 μm CMOS	477	1.5	NA	NA	3.3	–	–	Linear	YES
This Work	IRF510	1	7.42	89.9	5.2	20.3	Nonlinear	Nonlinear	Nonlinear	YES

*TC refers to thermal compensation.

## Conclusion

In this paper, a class E inverter with a thermal compensation unit has been presented. In the WPT, the class E inverter is more affected by temperature due to the presence of the active elements. The temperature variation effect has been considered in the class E inverter design including nonlinear C_ds_, C_gd_ and R_ON_ and it has been compensated to achieve a reliable power source in the biomedical implant. It has been found that temperature changes significantly affect inverter characteristics such as output power and efficiency. Therefore, a compensation circuit was proposed and added to the simple class E inverter. Simulations were performed and the results confirmed that the compensator enables significant improvements by maintaining almost constant power and efficiency (8.46 ± 0.14 W and 90.4 ± 0.2%) in the temperature range of − 60 to 100 °C. The thermal compensation class E inverter has been fabricated, and the simulation and theoretical results are validated with the measured results. The tested circuit results are in good and acceptable agreement with the simulation results. The obtained output power is 7.42 W with an efficiency of 89.9% at 25 °C.

## Data Availability

The calculated results during the current study are available from the corresponding author on reasonable request.
